# Enhanced cardiac expression of two isoforms of matrix metalloproteinase-2 in experimental diabetes mellitus

**DOI:** 10.1371/journal.pone.0221798

**Published:** 2019-08-28

**Authors:** Hye Won Lee, Sun Ju Lee, Min Young Lee, Mi Wha Park, Sang Sik Kim, Nari Shin, David H. Lovett, Sun Sik Bae, Jinhee Ahn, Jin-Sup Park, Jun-Hyok Oh, Jung Hyun Choi, Han Cheol Lee, Kwang Soo Cha, Taek Jong Hong, Sang Heon Song

**Affiliations:** 1 Biomedical Research Institute, Department of Internal Medicine, Pusan National University Hospital, Busan, Republic of Korea; 2 Department of Pathology, Hanmaeum Changwon Hospital, Changwon, Republic of Korea; 3 The Department of Medicine, San Francisco Veterans Affairs Medical Center, University of California, San Francisco, California, United States of America; 4 MRC for Ischemic Tissue Regeneration, Medical Research Institute, and Department of Pharmacology, Pusan National University School of Medicine, Yangsan, Republic of Korea; University of Louisville, UNITED STATES

## Abstract

**Background:**

Diabetic cardiomyopathy (DM CMP) is defined as cardiomyocyte damage and ventricular dysfunction directly associated with diabetes independent of concomitant coronary artery disease or hypertension. Matrix metalloproteinases (MMPs), especially MMP-2, have been reported to underlie the pathogenesis of DM CMP by increasing extracellular collagen content.

**Purpose:**

We hypothesized that two discrete MMP-2 isoforms (full length MMP-2, FL-MMP-2; N-terminal truncated MMP-2, NTT-MMP-2) are induced by high glucose stimulation in vitro and in an experimental diabetic heart model.

**Methods:**

Rat cardiomyoblasts (H9C2 cells) were examined to determine whether high glucose can induce the expression of the two isoforms of MMP-2. For the in vivo study, we used the streptozotocin-induced DM mouse heart model and age-matched controls. The changes of each MMP-2 isoform expression in the diabetic mice hearts were determined using quantitative real-time polymerase chain reaction (qRT-PCR). Immunohistochemical stains were conducted to identify the location and patterns of MMP-2 isoform expression. Echocardiography was performed to compare and analyze the changes in cardiac function induced by diabetes.

**Results:**

Quantitative RT-PCR and immunofluorescence staining showed that the two MMP-2 isoforms were strongly induced by high glucose stimulation in H9C2 cells. Although no definite histologic features of diabetic cardiomyopathy were observed in diabetic mice hearts, left ventricular systolic dysfunction was determined by echocardiography. Quantitative RT-PCR and IHC staining showed this abnormal cardiac function was accompanied with the increases in the mRNA levels of the two isoforms of MMP-2 and related to intracellular localization.

**Conclusion:**

Two isoforms of MMP-2 were induced by high glucose stimulation in vitro and in a Type 1 DM mouse heart model. Further study is required to examine the role of these isoforms in DM CMP.

## Introduction

Diabetic cardiomyopathy (DM-CMP) is defined as diabetes mellitus (DM)-induced ventricular dysfunction independent of concomitant coronary artery disease or hypertension [1,2]. The Framingham study demonstrated that heart failure in 6% of men and 12% of women can be attributed solely to DM [3]. The pathophysiology of this heart failure is complex and has been associated with enhanced reactive oxygen species (ROS) production, advanced glycation end product (AGE) formation, and protein kinase C signaling provoked by hyperglycemia, hyperinsulinemia, and hyperlipidemia [4]. Recent data indicate that defects in mitochondrial biogenesis also underlie the pathophysiology of diabetic cardiomyopathy [5–7]. Despite these recent advances in our understanding of the pathophysiology of diabetic cardiomyopathy, specific therapeutic options remain limited.

Previous reports have indicated that specific matrix metalloproteinases (MMPs) play important roles in several forms of cardiac disease, including ischemia-reperfusion (I/R) injury, post-infarction left ventricular remodeling, heart failure, and dilated cardiomyopathy [8–11]. Matrix metalloproteinase-2 (MMP-2) has been extensively studied in both experimental models and human cardiac disease and has been a therapeutic target in a limited number of clinical trials [12–14]. Until recently, both experimental and human studies were focused on the pathophysiologic roles of the full length MMP-2 isoform (FL-MMP-2), which has both extra- and intracellular proteolytic targets. The latter include sarcomeric troponin I, myosin light chain 1 and titin, with resultant diminishment of contractile function [15–17]. We recently reported on a novel intracellular, N-terminal truncated isoform of MMP-2 (NTT-MMP-2) generated by oxidative stress-mediated activation of an alternate promoter located in the first intron of the MMP-2 gene [18]. The NTT-MMP-2 isoform is localized to the mitochondrial intramembranous space and initiates cellular regulated necrosis by induction of the mitochondrial permeability transition [19].

We previously determined that the NTT-MMP-2 isoform is expressed in cardiomyocytes from mice with accelerated atherogenesis and myocardial infarction, as well as in the setting of aging [20]. Cardiac-specific transgenic expression of the NTT-MMP-2 isoform results in systolic failure associated with cardiomyocyte necrosis and inflammation [21]. In the present study we evaluated the expression of the MMP-2 isoforms in an experimental model of Type I diabetes mellitus and demonstrate an association between NTT-MMP-2 isoform expression, mitochondrial injury and systolic dysfunction.

## Materials and methods

### H9C2 cell culture experiments

Rat cardiomyoblasts (H9C2 cells) were purchased from American Type Culture Collections (ATCC, Rockville, MD, USA) and maintained in Dulbecco’s Modified Eagle’s Media (DMEM) (Gibco, Paisley, UK) containing 1.0g/L D-glucose supplemented with 10% fetal bovine serum (FBS) (Gibco). Cells were incubated at 37°C in a humidified incubator containing 5% CO_2_ and then cultured in the presence of 30mM D-glucose for 2 or 24 hrs and compared with controls maintained in normal glucose containing medium.

### Immunofluorescence of H9C2 cells

H9C2 cells were grown on sterilized glass coverslips in 6-well plates, washed with phosphate-buffered saline (PBS), fixed with 4% buffered paraformaldehyde for 20 minutes at 4°C and permeabilized in 0.1% Triton X-100/PBS for 10 minutes at 4°C. They were then incubated with FL anti-MMP2 antibody (ab3158, Abcam, Cambridge, UK) diluted 1:20 with 0.05% bovine serum albumin (BSA)/PBS or an affinity-purified NTT-MMP-2 specific antibody targeting the S1’ substrate binding loop at 5 μg/ml in 0.05% BSA/PBS [18,22]. Both primary antibody incubations were performed at 4°C overnight.

Coverslips were then incubated with fluorescein isothiocyanate (FITC)-conjugated secondary antibody (Molecular Probes) for FL-MMP-2 (1:1,000, Alexa fluor^®^ 488 rabbit anti-mouse IgG), and NTT-MMP-2 (1:800, Alexa fluor^®^ 488 rabbit anti-goat IgG) at room temperature for 30 minutes. Cell nuclei were stained with DAPI (4,6-diamidino-2-phenylindole) (Molecular Probes, Oregon, USA) at 1:20,000 for 10 minutes and with 20 nM of Mitotracker Red (M7512, Invitrogen, Korea) for 25 minutes to visualize mitochondria. After mounting coverslips on microscopy glass slides with fluorescent mounting medium (Dako, Carpinteria, CA, USA), images were obtained with a Leica TCS-SP8 confocal microscope (Leica, Mannheim, Germany). Immunofluorescence intensities as well as Mitotracker staining which were normalized per each cell number (DAPI signal) were measured with the Image J program (NIH, USA) and were presented with mean ± standard deviation. Every experiment was repeated at least 3 times.

### Quantitative real-time reverse transcriptase-polymerase chain reaction (RT-PCR)

MMP-2 mRNA isoform levels were assessed by quantitative RT-PCR. For the cardiac tissue, weight of each sample was ranged from 0.08g to 0.1g. Total RNA was extracted using 1ml of TRIzol reagent (Thermo Fisher Scientific Inc, CA, USA) for each sample according to the manufacturer’s instructions. After centrifuge, we collected 400μl of supernatant to 1.5ml tube, and then RNA was extracted. Acquired total RNA was quantified with Synergy^TM^ H1 hybrid reader (BioTek Instruments Inc, Winnosk, VT, USA). All tissues were normalized after repeated total RNA measurement, and total RNA (5 μg) was used to synthesize cDNA using oligo-dT primers and Moloney Murine Leukemia Virus Reverse Transcriptase (MMLV RTase, Promega, Madison WI, USA). The reaction was performed at 42°for 1 hour. The primers used for RT-PCR are described in Table 1. Amplification was conducted over 40 cycles (95°C for 15 seconds; 60°C for 45 seconds; and 72°C for 1 minute) using Fast Start Universal SYBR Green Master Mix (Rox dye, Roche) and an ABI 7500 Real-time PCR system (Applied Biosystems). β-actin was used as internal control and all products were verified by melting curve analysis (95°C 15 sec, 60°C 15sec, 95°C 15sec). Gene normalization was performed by comparing with the expression of the other internal control gene between different samples, and fold-changes were determined using the 2^-ΔΔCT^ method. Experiments were repeated at least 3 times to see similarity of the results.

**Table 1:** Primer sequences used for quantitative real-time polymerase chain reactions.

Genes	Forward (5’→3’)	Reverse (5’→3’)
Full length MMP-2 (rat)	TCGCCCATCATCAAGTTCCC	GGGCAGCCATAGAAGGTGTT
N-terminal truncated MMP-2 (rat)	GCTGTATGTCCTGTCGCT CAA CT	GGGCAGCCATAG AAGGTGTT
Full length MMP-2 (mouse)	GACCTCTGCGGGTTCTCTGC	TTGCAACTCTCCTTGGGGCAGC
N-terminal truncated MMP-2 (mouse)	GTGAATCACCCCACTGGTGGGTG	TTGCAACTCTCCTTGGGGCAGC
β-actin (mouse control)	CTCTCTTCCAGCCTTCCTTCC	CTCCTTCTGCATCCTGTCAGC

### Murine diabetic cardiomyopathy model

Ethical aspects of the study protocol (PNUH 2016–098) were reviewed and approved by the Pusan National University-Institutional Animal Care and Use Committee (PNU-IACUC). Eight-week-old C57/BL6j male mice were obtained from Koatech Technology Corporation (Korea) and randomly allocated to a diabetic or a control group (8 mice/group). Type I diabetes mellitus was induced by injecting streptozotocin intraperitoneally (40 mg/kg in citrate buffer, pH 4.5, Sigma-Aldrich) for 5 consecutive days. Blood glucose levels were measured by tail puncture using a blood glucometer 1 week after the final injection. Body weights were measured weekly and blood glucose concentrations were monitored in the first 2 weeks and every four weeks thereafter to confirm sustained hyperglycemia. Hyperglycemia was defined as a blood glucose level >300 mg/dL. Hearts were harvested under isoflurane anesthesia after perfusion with 4°C PBS at 12 weeks post-injection. Those mice were sacrificed by rapid cardiectomy after given ketamine/xylazine (80mg/kg IP for ketamine, 10mg/kg IP for xylazine), and tissue harvest as described below. Cardiac apices from 8 animals were retained for quantitative RT-PCR and electron microscopic examination (4 animals for gelatin zymography study). Remaining portions were fixed in 10% neutralized formalin for the immunohistochemistry (IHC) study.

### Gelatin zymography

Zymography was performed to determine MMP-2 activities in heart tissues. Cardiac protein was extracted using lysis buffer (25mM Tris-HCl, pH 7.5, 100mM NaCl, 1% NP-40) 120μg per 30mg of heart tissue, and then centrifuged at 16000g for 10 min at 4°C. Saved supernatant was measured with Bradford protein assay. A total of 50μg of protein was equally loaded in each sample. Gelatinase activity was determined by electrophoresis on 8% SDS-PAGE gels containing 0.1% (w/v) gelatin. Gels were incubated for 40 min in zymogram renaturing buffer (Komabiotech, Korea) at room temperature, washed in distilled water, and then incubated for 36 hours in zymogram developing buffer (Komabiotech, Korea) at 37°C according to the manufacturer’s instructions. MMP gelatinolytic activities were detected after staining the gels with Coomassie Brilliant Blue R250 as clear bands on a blue background.

### Immunohistochemistry analysis

Formalin-fixed, paraffin-embedded tissue blocks were sectioned at 3μm, deparaffinized in xylene, hydrated in a graded ethanol series, rinsed with distilled water, and washed with PBS. Sections were stained with Hematoxylin-Eosin stain (H&E), Masson’s Trichrome (MT), and Periodic acid-Schiff (PAS) stain. IHC was performed using the avidin-biotin complex technique using VIP (Vector labs, USA) as chromogen. Sections were then incubated for 30 min in methanol containing 3% H_2_O_2_ (to block endogenous peroxidase activity), washed with PBS, and incubated overnight at 4°C with primary antibody for pre-diluted FL anti-MMP-2 antibody (FL-MMP2, ab3158, Abcam, UK) or NTT-MMP-2 (affinity purified, 5 ug/ml). Immunolabeling was detected using biotinylated immunoglobulins followed by Vector stain ABC complex (Vector). All slides were counterstained with methyl green. Quantitative IHC analysis was performed using the Aperio Positive Fixel Count Algorithm with an Aperio Scan Scope analyzer (Leica, Germany), and the Image J program (NIH, USA). Randomly selected 5 sections in high resolution (x400 magnification) of 3 slides from control group and from 8 slides of diabetic group were analyzed. They were presented as % signal area (normalized to the tissue mass of each sections) and analyzed.

### Terminal deoxynucleotidyl transferase dUTP nick end labeling (TUNEL) staining

To quantify nuclear DNA damage levels, cells were stained using the immunofluorescent TUNEL technique (kit S7110; Millipore, Darmstadt, Germany). Slides were mounted using 70% glycerol in PBS mounting solution. Cells exhibiting positive nuclear staining, indicative of DNA fragmentation, were visualized directly under a 40X confocal microscope (Leica, Germany). The number of TUNEL-positive cells were counted in at least 3 random fields per section.

### Electron microscopic analysis

LV apex tissues or H9C2 cells were pre-fixed with 2.5% glutaraldehyde (4°C, phosphate buffer, pH 7.2), and then post-fixed with 1% osmium tetroxide in the same buffer. Samples were then dehydrated using an ethanol series and embedded in epoxy resin (Epon 812). Thick sections (1μm) were stained with 1% toluidine blue for light microscopy, and thin sections (50~60nm) were prepared using an ultramicrotome (EM UC7, Leica), and double stained with uranyl acetate and lead citrate. Thin sections were examined by transmission electron microscopy (JEM-1200EXⅡ, JEOL). Subcellular structures of organelles were reviewed and degrees of mitochondrial abnormality were assessed semi-quantitatively using the scoring system described in Table 2.

**Table 2:** The electron microscopy based scoring system used to assess mitochondria damage.

Damage score	Description
Mild	Number of mitochondria with ruptured membrane ≤25% at randomly selected 5 samples from 10K magnification
Moderate	Number of mitochondria with ruptured membrane >25% and ≤75% at any samples from randomly selected 5 samples from 10K magnification
Severe	Number of mitochondria with ruptured membrane ≥75% at any sample from randomly selected 5 samples from 10K magnification

### Transthoracic echocardiography

Cardiac function was assessed by transthoracic echocardiography under anesthesia. Three mice from the control group and six mice from the diabetic group were selected for echocardiography. Chest hair was removed with a hair removal gel before image acquisition and a warming pad was used to reduce heat loss. For each mouse, parasternal long-axis and short-axis views and M-mode images were obtained at the left ventricular (LV) papillary muscle level. We used a GE Vivid7 and a 10 MHz linear probe for the cardiac examinations, and images were acquired using a frame rate of 400 f/s, a minimal depth of 1.5 cm, and sweep speed of 100 mm/s. For echocardiographic measurements of LV systolic function, LV interventricular septal thickness (IVS), LV internal dimensions (LVID), and posterior wall thicknesses (PW) at diastole and systole (IVSd, LVIDd, PWd, and IVSs, LVIDs, PWs, respectively) were measured using M-mode images. LV ejection fraction (EF) and LV fractional shortening (FS) were calculated using the following formulae.

$$EF\;(\%)=100\;x\;\left[\left(LVIDd^{3}‐LVIDs^{3}\right)/LVIDd^{3}\right]$$

FS(%)=100x[(LVIDd‐LVIDs)/LVIDd]

### Statistical analysis

The analyses were performed using GraphPad Prism 5.0 (GraphPad Software). Results are presented as mean ± standard deviation. The Mann-Whitney U test or the Kruskal-Wallis test were used for intergroup comparisons, as appropriate. Statistical significance was accepted for p values < 0.05.

## Results

### High glucose stimulation induces both MMP-2 isoforms in H9C2 cardiomyoblasts

**Fig 1:**
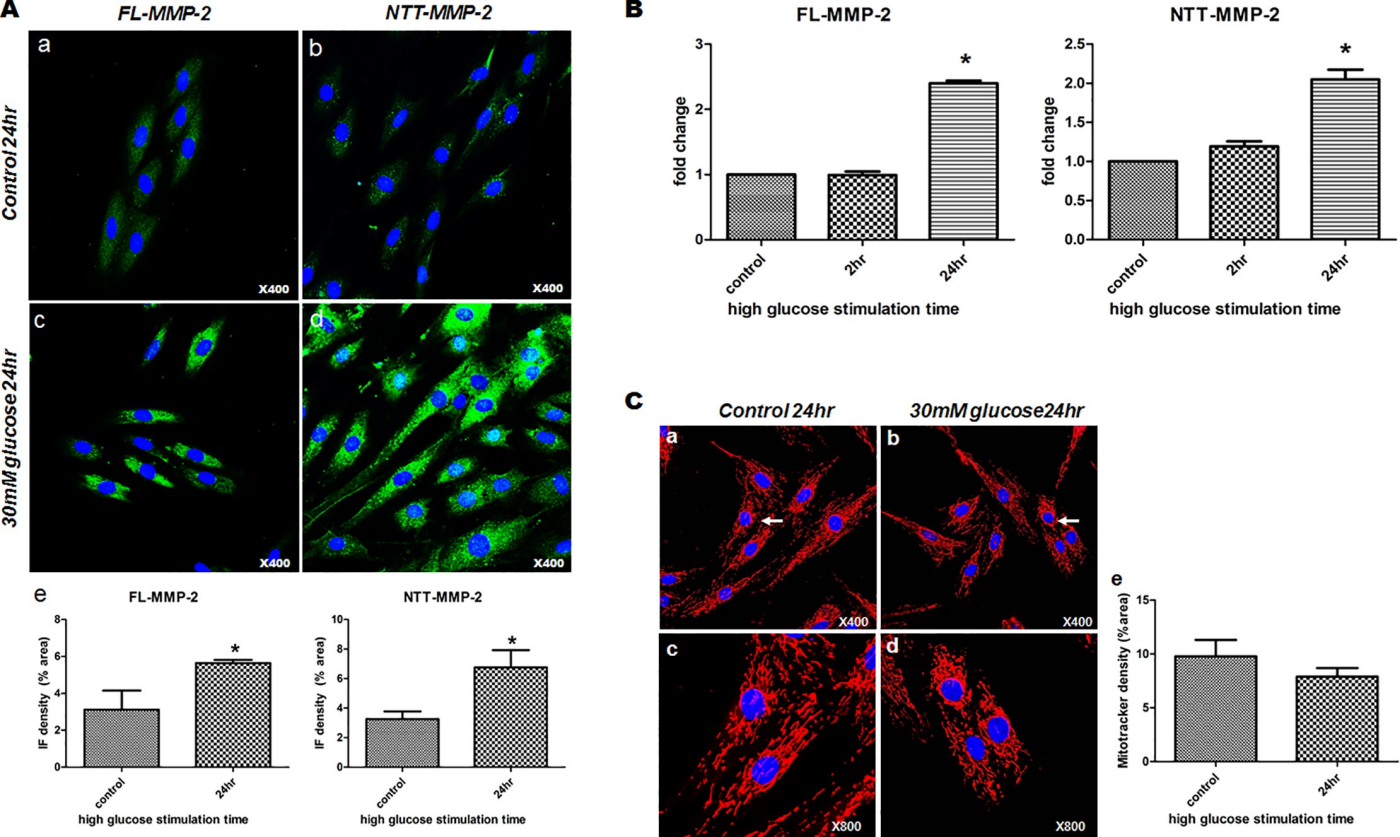
Expressions of the two MMP2 isoforms in 30mM glucose stimulated H9C2 cells. (A) Immunofluorescence staining of H9C2 cells stimulated for different times. a: FL-MMP-2 staining after exposure to normal glucose for 24hours (x400) showing minimal staining in cytosol, b: NTT-MMP-2 staining after exposure to normal glucose for 24 hours (x400) was negative, c: FL-MMP-2 staining after exposure to 30 mM glucose 24 hours (x400) showing FL-MMP-2 was highly expressed throughout cytosol, d: NTT-MMP-2 staining after exposure to FL-MMP-2 30mM glucose for 24hours (x400) was localized to punctate lesions throughout cytosol and near nuclei. e: Immunofluorescence density analysis of FL- and NTT-MMP-2 in H9C2 cells using the Image-J program showing increased FL- and NTT-MMP-2 staining after 30mM glucose stimulation as compared with normal glucose stimulation. Each IF signal was normalized to cell number of DAPI signal. (B) Real-time quantitative PCR of the two isoforms of MMP-2 after normal or 30mM glucose stimulation. mRNA levels of FL- and NTT-MMP-2 isoforms were significantly increased at 24hours of 30mM glucose exposure, as compared with after exposure for 0 (baseline) or 2 hours. (C) Mitotracker results for H9C2 cells treated with different glucose concentrations. a, c: low (x400) and high (x800) magnification photographs of H9C2 cells exposed to normal glucose showing normal rod shapes and mitochondria evenly distributed in cytosol. b, d: low (x400) and high (x800) magnification photographs of H9C2 cells exposed to 30mM glucose exposure showing centrally located fragmented, short mitochondria, around nuclei (white arrows). e: Mitotracker density analysis showed a trend of decreased density in high glucose group compared to control group. Each IF signal was normalized to cell number of DAPI signal.

Immunofluorescence staining of H9C2 cells showed FL-MMP-2 and NTT-MMP-2 levels were elevated by high glucose stimulation for 24 hrs as compared to the non-treated control group (FL-MMP-2: 5.65 ± 0.17% in treated group vs. 3.12 ± 1.03% in control group, p = 0.029, NTT-MMP-2: 6.75 ± 1.18% in treated group vs. 3.26 ± 0.52% in control group, p = 0.042). FL-MMP-2 staining was observed diffusely throughout the cytosol. In contrast, NTT-MMP-2 immunofluorescence staining analysis showed a more punctate expression pattern consistent with a previously reported mitochondrial localization [19]. (Fig 1, panel A, a-d). Furthermore, high glucose stimulation induced FL-MMP-2 mRNA expression time-dependently in H9C2 cells as determined by quantitative RT-PCR (1.00 ± 0.07 fold at 2 hr; 2.4 ± 0.07 fold, *p* = 0.001 at 24 hr vs. non-treated controls). NTT-MMP-2 mRNA expression was also significantly increased by high glucose stimulation (1.19 ± 0.09 fold at 2 hr; 2.05 ± 0.17 fold, *p* = 0.014, at 24 hr vs. non-treated controls) (Fig 1, panel B). Mitotracker staining showed that high glucose stimulation numerically reduced overall mitochondria numbers which was associated with fragmentation in H9C2 cells. However, there was no statistical difference between two groups (9.78 ± 2.15% in control group vs. 7.9 ± 1.59% in treated group, p = 0.571) (Fig 1 panel C).

**Fig 2:**
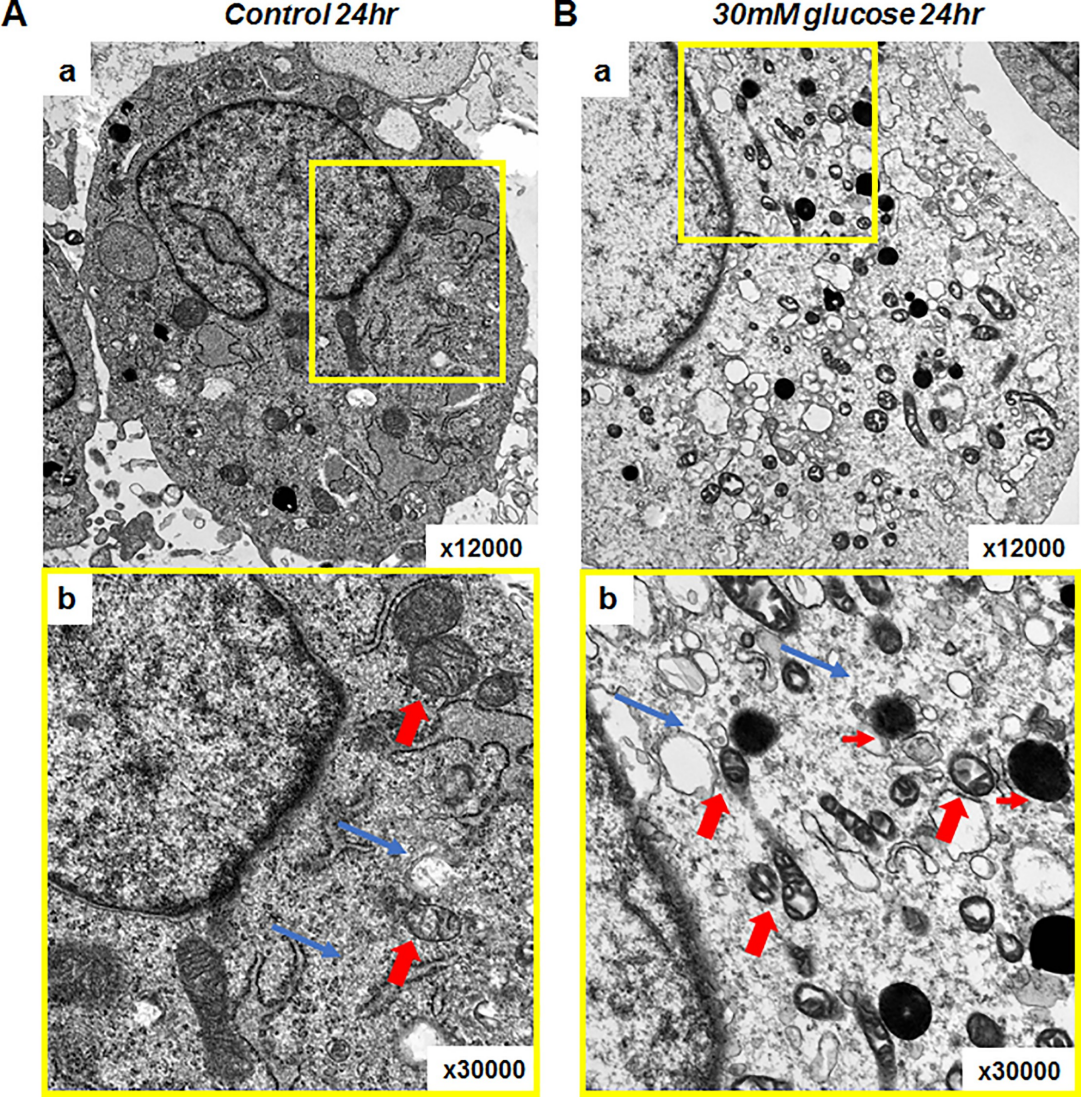
Electron microscopic evaluation of H9C2 cells showing mitochondria were damaged by exposure to 30mM glucose. (A) H9C2 cells exposed to normal glucose for 24 hours. a: H9C2 cells exposed to normal glucose for 24 hours (x12,000), b: Close up of a (yellow box) shows normally sized, well organized mitochondria (thick red arrows) and intracellular organelles. Materials and myofilaments in cytosol were evenly distributed (thin blue arrows) (x30,000). (B) H9C2 cells exposed to 30mM glucose for 24 hours. a: Many small, abnormal mitochondria were distributed throughout cytosol. b: Abnormal small, mitochondria lacking cristae. Abnormal vacuoles (thin red arrows) and sparse cytosolic materials including myofilaments (thin blue arrows).

Transmission electron microscopy of H9C2 cells confirmed and extended the results seen at the light microscopy level with Mitotracker. As shown in Fig 2, culture of H9C2 cells in high glucose medium for 24 hours resulted in mitochondrial swelling, loss of organized cristae structure and fragmentation consistent with the mitochondrial permeability transition. In addition, there was evidence for mitochondrial autophagy (mitophagy). Thus, culture of cardiomyoblast H9C2 cells in high glucose medium induces MMP-2 isoform expression associated with light and transmission electron microscopy findings characteristic of the mitochondrial permeability transition.

### Diabetic mice exhibit decreased LV systolic function in the absence of structural changes

**Fig 3:**
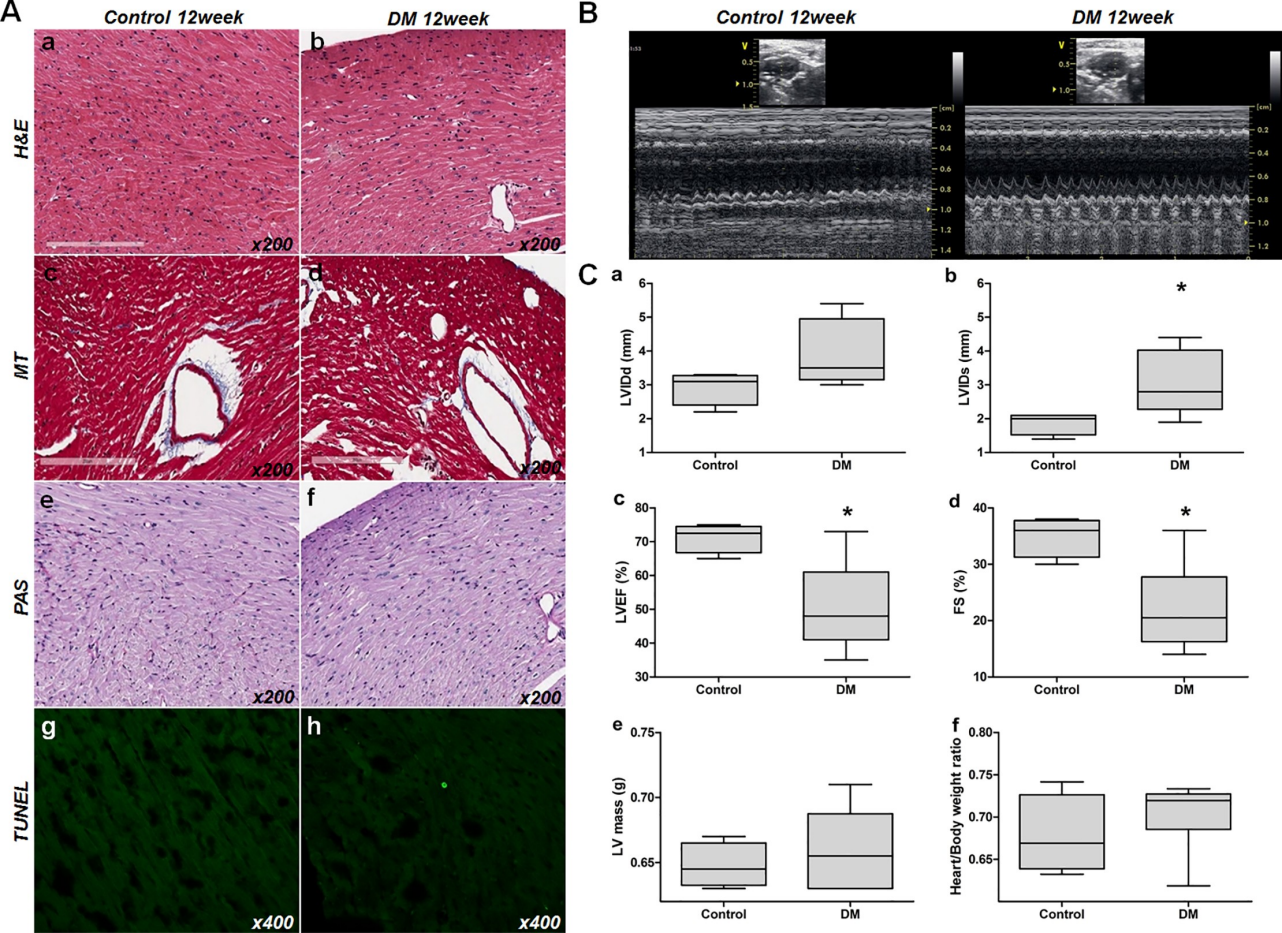
Histologic and functional changes in diabetic mouse hearts. (A) Optical microscopic findings. a,b: H&E stained control and DM mouse sections. c, d: MT stained control and DM mouse sections. e, f: PAS stained control and DM mouse sections. g, h: TUNEL stained control and DM mouse sections. No evidence of tissue injury, such as inflammation, sarcomere disruption, apoptosis, necrosis, or fibrosis (x200), was observed. (B) Transthoracic echocardiogram of control (left panel) and the STZ-induced diabetic mice hearts (right panel). LV internal dimensions at diastole; LVIDd, LV internal dimension at systole; LVIDs, LV ejection fraction; LVEF, fractional shortening; FS. (C) Analysis of echocardiogram measurements. a: LVIDd (mm), b; LVIDs (mm), c; LVEF (%), d: FS (%), e: LV mass (g), f: heart/total body weight ratio. [abbreviations: LVIDd, left ventricular internal dimension at diastole; LVIDs, left ventricular internal dimension at systole; LVEF, left ventricular ejection fraction; FS, fractional shortening].

Echocardiography was performed to evaluate cardiac functional changes 12 weeks following induction of Type 1 diabetes mellitus and compared to age-matched litter mate controls. Mean body and heart weights were similar between the two groups (diabetic group 27.17 ± 1.72 g and 0.19 ± 0.02 g, vs. control 26.0 ± 3.37g and 0.18 ± 0.02g, p = 0.557 and p = 0.191, respectively). Mean heart to body weight ratios were comparable in both groups (0.70 ± 0.04% vs. 0.68 ± 0.05%, p = 0.409). Hematoxylin and eosin (H&E) staining showed no definite inflammatory cell infiltration, cardiomyocyte sarcomeric disorganization or necrosis in the diabetic group (Fig 3, panel A, a,b). Masson’s trichrome (MT) staining showed no evident increase in fibrosis in the diabetic group as compared to controls (Fig 3, panel A, c, d). Periodic acid-Schiff (PAS) staining showed no definite increase in advanced glycation end product deposition or coronary artery hyalinization in the diabetic hearts as compared to controls. (Fig 3, panel A, e, f). Only rare TUNEL-positive cardiomyocytes were observed in the diabetic hearts and this was not significantly increased as compared to controls. (Fig 3, panel A, g, h).

Although we did not observe any histological changes characteristic of diabetic cardiomyopathy in the diabetic group at 12 weeks, functional changes were evident as compared to the control group. Diabetic hearts had significantly larger LV systolic dimensions and lower LVEF and FS than control hearts (LV internal dimension at systole; 1.88 ± 0.33mm vs. 3.03 ± 0.95mm, *p* = 0.03, LVEF; 71.25 ± 4.35% vs. 50.67 ± 13.5%, *p* = 0.012, and FS; 35 ± 3.56% vs. 22.17 ± 7.88%, *p* = 0.009, respectively) (Fig 3, panel B, C). No significant intergroup differences were observed between LV mass, LV internal dimensions at diastole, or interventricular septal thickness, which represent LV structural remodeling. However, there was increased collagen accumulation analyzed by MT staining at 24 weeks (S1 Fig).

### MMP-2 isoform expression is increased in diabetic hearts with distinctive patterns

**Fig 4:**
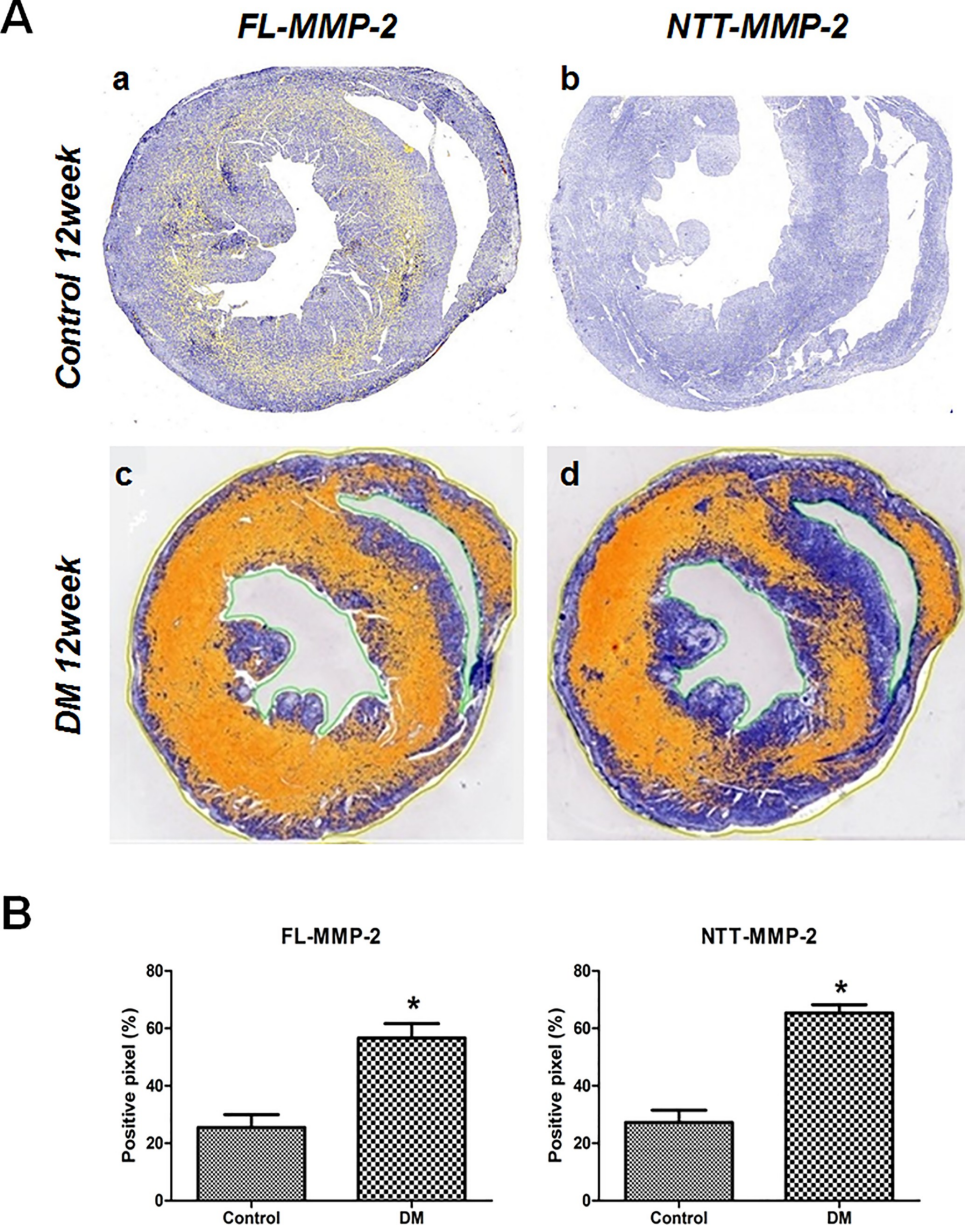
Immunohistochemical stain for two isoforms of MMP-2. (A) IHC expression of the two MMP-2 isoforms in transverse sectional images (papillary muscle level) of control and STZ-induced diabetic mouse hearts. MMP-2 expressing areas were colored using yellow or red dots using a staining intensity algorithm. a: FL-MMP-2 staining was minimal at 12 weeks after streptozotocin injection control mice. b: NTT-MMP-2 was not expressed in non-diabetic control mice. c: FL-MMP2 was expressed strongly in the near whole area of the section in diabetic mouse hearts. d: NTT-MMP-2 was also strongly expressed and its expression pattern was similar to that of FL-MMP-2 in diabetic mouse hearts. (B) Stained pixel numbers were counted using the Aperio Positive Pixel Count Algorithm. IHC pixel density analysis of FL- and NTT-MMP-2 in transverse section images showed greater expressions of both MMP-2 isoforms in diabetic mouse heart compared to the control group (*p*<0.05) (by Aperio Image Scope v12.3.2.8013, Leica Biosystems Pathology Imaging, USG).

As shown in Fig 4, panel A, a, there is a low, but detectable amount of FL-MMP-2 IHC staining of control cardiac cross sections in both the right and left ventricles. In contrast the NTT-MMP-2 isoform was not detectable by IHC in the control ventricles (Fig 4, panel A, b). At 12 weeks following induction of Type I diabetes mellitus there are significant increases in the IHC staining for both isoforms (Fig 4, panel a, c, d). Interestingly, staining for both isoforms was most prominent within the myocardium, with relative little expression on the epicardial and endocardial surfaces.

Quantitative pixel counting of MMP-2 isoform IHC staining showed significantly greater positive staining rates for both MMP-2 isoforms in the ventricles of diabetic mice: FL-MMP-2: 56.6 ± 5.0% vs. 25.5 ± 4.5%; NTT-MMP-2; 65.4± 2.9% vs. 27.2 ± 4.2%; for controls and diabetic mice, respectively, *p*<0.001. Fold expression levels of the MMP-2 isoforms are shown in Fig 4, Panel B.

**Fig 5:**
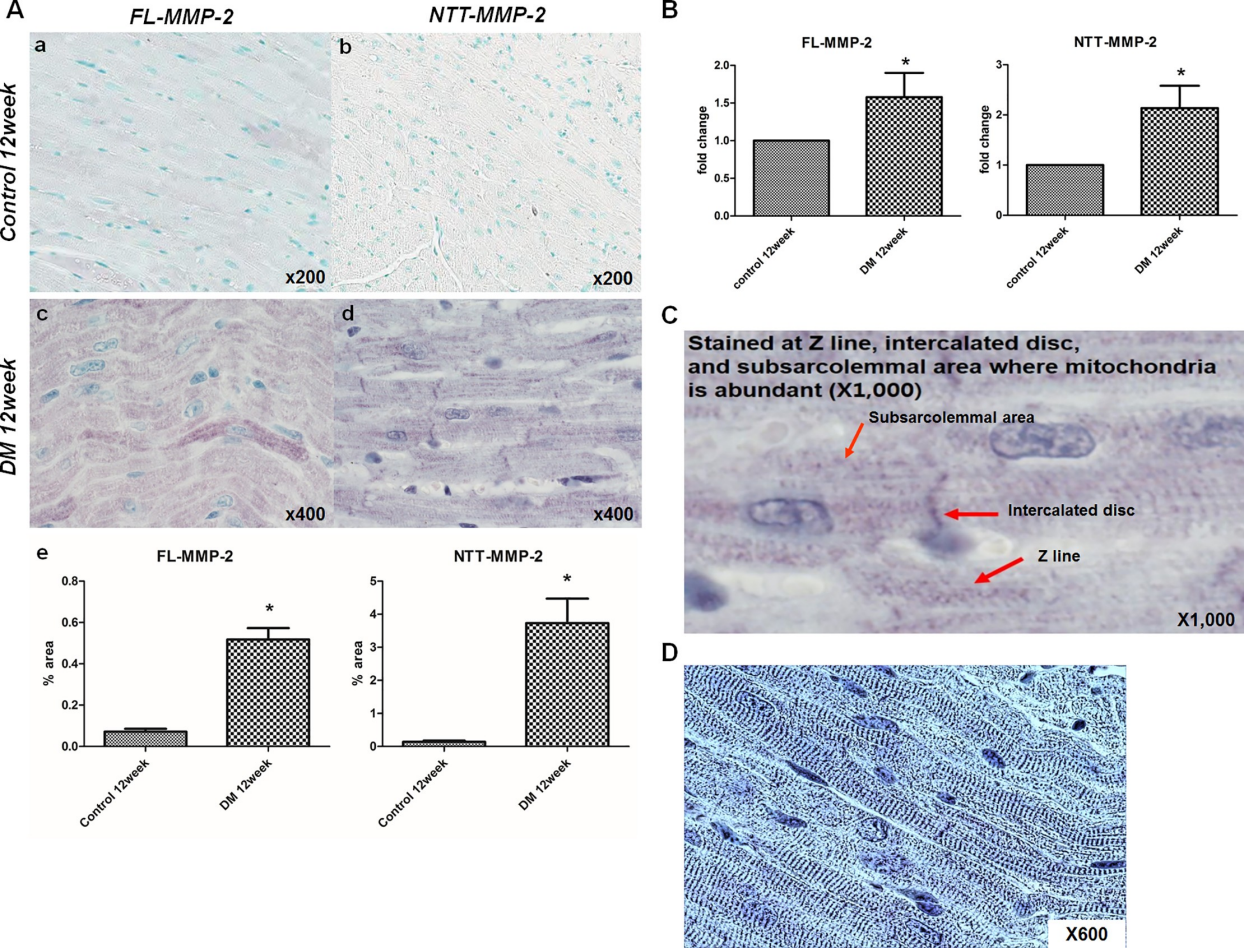
High resolution analysis of MMP-2 isoform staining, and real-time quantitative PCR analysis. (A) Optical microscopic findings. The expression of the two MMP-2 isoforms as determined by immunohistochemistry (IHC; Original magnification x200, x400). a, b: The expressions of both isoforms were minimal in control mice. c, d: FL-MMP-2 staining showed FL-MMP-2 was mainly localized at sarcomeres, while NTT-MMP-2 staining shows NTT-MMP-2 was localized at Z- lines, nuclei, and subsarcolemmal areas in diabetic mice (longitudinal section). e: Quantitative analysis of both isoforms staining shows significantly increased staining of both MMP-2 isoforms in diabetic group compared to control group. (B) Real-time quantitative PCR of the two isoforms of MMP-2 in control and diabetic mouse hearts. FL- and NTT-MMP-2 isoform transcripts were upregulated strongly in diabetic heart tissues. (C) High resolution (x1000) images showed that NTT-MMP-2 expressed the specific area within cardiac muscle cells such as Z-line, intercalated disc and subsarcolemmal area where the mitochondria are abundant (red arrows). (D) In contrast conversed image, these findings could be identified (blue lines) consistent with the previous results (^18^,^19^,^22^).

The results of higher powered microscopy analysis of IHC staining of control and diabetic hearts for the FL-MMP-2 and NTT-MMP-2 isoforms are summarized in Fig 5. There is low, but detectable expression of the FL-MMP-2 isoform in ventricles of control mice, while the NTT-MMP-2 isoform was not detectable in controls (Fig 5, Panel A, a, b). At 12 weeks following induction of diabetes mellitus, there were significant increases in IHC staining for both isoforms (Fig 5, Panel A, c, d). Quantitative analysis revealed that 7- and 26-folds increased expression of FL-MMP-2 and NTT-MMP-2, respectively (FL-MMP-2; 0.07±0.06% vs. 0.52±0.24%, p<0.0001, NTT-MMP-2; 0.14±0.15% vs. 3.73±3.62%, p = 0.0026). IHC staining of the FL-MMP-2 isoform revealed decoration of sarcomeres, while the NTT-MMP-2 isoform staining was particulate in nature, consistent with our previously reported mitochondrial localization [19]. Real-time quantitative PCR analysis showed increased expression of both isoforms of MMP-2 at 12 week of diabetic induction (FL-MMP-2: 1.56 ± 0.66 fold in diabetic group vs. control group, *p = 0*.*02*, NTT-MMP-2: 2.14 ± 1.18 fold in diabetic group vs. control group, *p = 0*.*09*) (Fig 5, Panel B). Higher power light microscopic examination of the distribution of cardiomyocyte NTT-MMP-2 staining revealed a complex intracellular distribution. There was evidence of IHC staining for this isoform on the Z-lines of individual cardiomyocytes, as well as in the subsarcolemmal area and the intercalated discs (Fig 5, Panel C, D).

**Fig 6:**
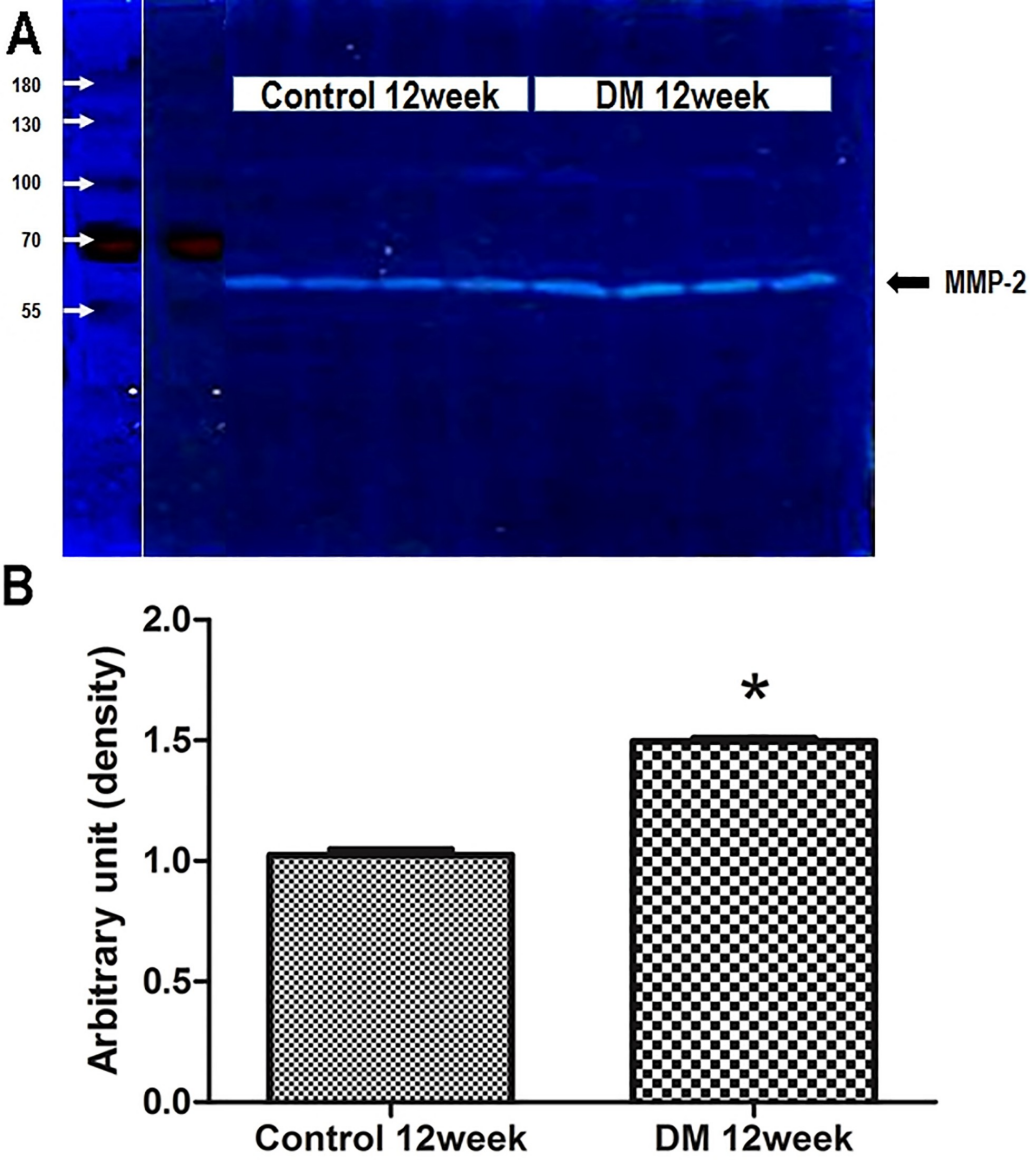
Enzymatic activities measured by zymography. (A) Gelatin zymography of the non-diabetic control and diabetic mouse hearts. Zones of lysis denote gelatin enzymatic activity for MMP-2 (68kDa). (B) MMP-2 enzymatic activity was greater in diabetic heart tissues than in controls. Band intensities were quantified by measuring pixel intensities. MMP-2 enzymatic activity was greater in diabetic heart tissues than in non-diabetic control hearts (1.03±0.04 vs. 1.49±0.03, p = 0.029).

Quantitative gelatin zymography of ventricular extracts confirmed increased expression of MMP-2 at 12 week following induction of diabetes mellitus. As shown in Fig 6, there is an approximate 1.5-fold increase in total gelatinase activity in ventricular extracts of diabetic mice as compared to age-matched litter mate controls. As the resolution of gelatin zymography is insufficient to distinguish between the 68 kDa FL-MMP-2 and the 65 kDa NTT-MMP-2 isoforms, the data are given as total gelatinase activity.

### The degree of NTT-MMP-2 expression and mitochondrial structural damage

**Fig 7:**
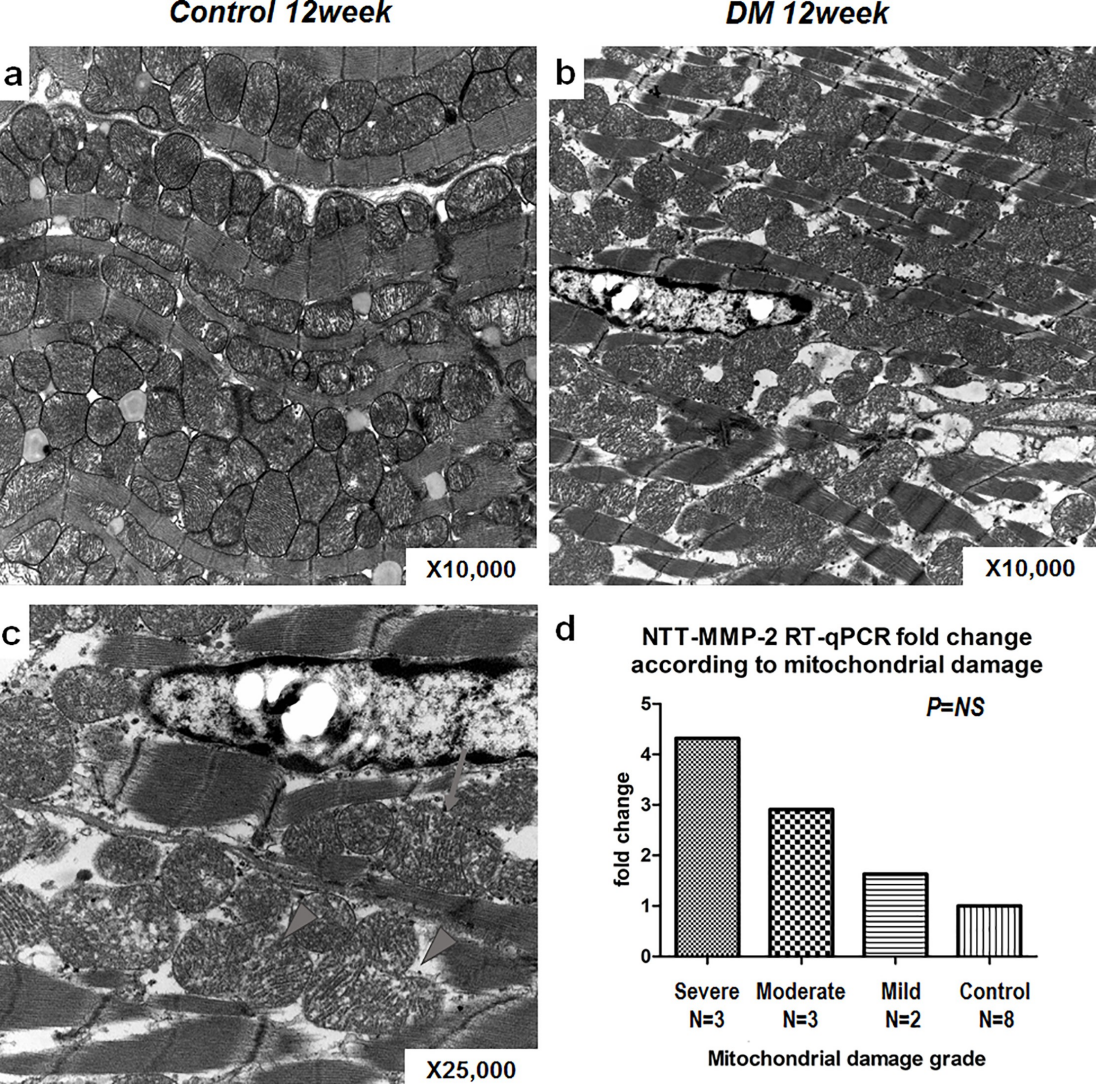
The relationship between NTT-MMP-2 expression and degree of mitochondrial damage. a~d: Electron microscopic analysis of control and diabetic heart tissues. a: This image shows well-organized sarcomeres and mitochondria in control heart tissues (x12000). b: The image shows small, fragmented mitochondria among sparse and widened sarcomeres in diabetic mouse heart (x10000). c: Image showing damaged, ruptured mitochondria with discharges (arrow) and fissuring of mitochondria (arrowheads) at high resolution in a diabetic mouse heart (x25000). d: The degrees of mitochondrial damage (as assessed using our semi-quantitative scoring system) were related to the NTT-MMP-2.

Electron microscopy was used to score in a semi-quantitative manner the degree of mitochondrial structural abnormalities as in Table 2 and compared to qPCR quantitation of MMP-2 isoform transcript abundance. The results of these studies are summarized in Fig 7. As compared to controls, mitochondria from hearts at 12 weeks following induction of diabetes mellitus displayed to typical morphologic changes of the mitochondrial permeability transition, with swelling, loss of mitochondrial membrane integrity, dissolution of cristae and mitochondrial rupture and fragmentation. The more severe the degree of mitochondrial damage was, the fold-increase in NTT-MMP-2 transcript was bigger but without statistical significance due to low number of animals.

## Discussion

The principal findings of this study are that the FL-MMP-2 and NTT-MMP-2 isoforms of MMP-2 are induced by hyperglycemic stimuli in both *in vitro* and *in vivo* diabetic cardiac models. This is the first report of the expression of the FL-MMP-2 and NTT-MMP-2 isoform in a model of Type I diabetic cardiomyopathy. As was reported in a previous study of an I/R cardiac injury model by Lovett, et al.[18], the two MMP-2 isoforms were associated with distinctive intracellular structures in cardiomyocytes. FL-MMP-2 was localized mainly to cardiomyocyte sarcomeres, whereas NTT-MMP-2 was concentrated in the subsarcolemmal space, where mitochondria are abundant. In addition, we observed NTT-MMP-2 immunohistochemical staining within Z-lines, intercalated discs, and nuclei. These non-mitochondrial localizations of the NTT-MMP isoform appear to be distinctive to the diabetic cardiomyopathy model, as we previously found that cardiac specific transgenic expression of the NTT-MMP-2 within normal mice resulted in only mitochondrial localization [18]. Mitochondrial morphological changes characteristic of the mitochondrial permeability transition were observed in diabetic cardiomyocytes and the degrees of mitochondrial damage were found to be positively correlated with the degree of NTT-MMP-2 expression as determined by qRT-PCR.

The histological characteristics of human diabetic cardiomyopathy are only partially present in the hearts examined in this study. The well-known histological characteristics of established cardiomyopathy in the Type 1 diabetes mellitus are cardiomyocyte hypertrophy, interstitial collagen deposition, and LV fibrosis [4,23]. In addition, in early diabetic cardiomyopathy there is mitochondrial dysfunction in terms of energy production, and increased oxidative stress. Structurally there is decreased LV wall thickness, increased LV diameter and volume, diminished LV ejection fraction and cardiac output. Further, an increased E/A ratio are evident, which is consistent with combined systolic and diastolic dysfunction [24–26]. The insulin resistance and hyperinsulinemia which occur in humans with prediabetic metabolic syndrome are major pathophysiological factors in the type 2 diabetic cardiomyopathy [27]. These factors are responsible for the collagen deposition, LV fibrosis, and predominant diastolic dysfunction observed in type 2 diabetic cardiomyopathy, whereas systolic dysfunction without predominant fibrosis is observed in the type 1 diabetic heart [28]. The *in vivo* echocardiographic and histologic data obtained during this study showed significant systolic dysfunction and chamber dilation preceded fibrotic change, which was not observed until 24 weeks. These results are in line with those of previous studies on type 1 DM, as described above. The intracellular pathologies described in this report, particularly of the mitochondria may account for the chamber dilation and cardiac systolic dysfunction prior to the development of apoptosis and fibrosis. Our results indicate the MMP-2, especially intracellularly located isoforms of MMP-2, may be involved in the primarily functional changes seen in early type 1 diabetic cardiomyopathy.

Experimental evidence shows that MMP-2 is activated in I/R injury and congestive heart failure, and that pharmacological inhibitors of MMP activity or MMP-2 neutralizing antibody prevent LV remodeling and aid recovery of mechanical function [29,30]. Moreover, myocardial-specific overexpression of constitutively active FL-MMP-2 in mouse heart induced severe LV remodeling, systolic dysfunction, sarcomere disruption, and mitochondrial damage even in the absence of significant external injury [31,32]. In addition to the proteolytic removal of pro-peptide domain MMP-2 in the peri-cellular environment [33], MMP-2 can also be activated intracellularly by S-glutathionylation or phosphorylation [34,35], or even by alternative splicing of its 1^st^ intron by oxidative stress [18]. The FL-MMP-2 isoform has been shown to degrade Troponin I and led to impair cardiac contractile function in I/R Injury [15], and myosin light chain-1 and the cytoskeletal proteins desmin, spectrin, α-actinin, and titin have been shown to co-localize with MMP-2 [36]. Our study showed that the intracellular FL-MMP-2 isoform was localized to the sarcomeres, which suggests intracellular MMP-2 is also induced in diabetic cardiomyopathy and that it caused cardiac contractile dysfunction during early stage DM CMP without inducing ECM fibrosis or LV remodeling. We also found that NTT-MMP-2 was also induced in vivo and in vitro model of diabetes. Considering that our previous study [18] demonstrated NTT-MMP-2 was localized to the mitochondria, the mitochondrial damage observed in the present study may have been induced by NTT-MMP-2 activation. This mitochondrial change might explain the functional changes in our early DM CMP model. Future studies on the role of the intracellular MMP-2 isoforms may result in improving our understanding diabetes-induced cardiac pathophysiologic derangement and aid the identification of new therapeutic targets in oxidative stress-induced cardiac diseases, including DM-CMP.

There are some limitations in the present study. Especially, diabetic murine model in this study did not show the prominent pathological histologic changes compatible with cardiomyopathy, and further studies using other diabetic murine models are needed to understand and uncover the role of MMP-2 in diabetic cardiomyopathy. In addition, because it is unclear whether intracellular isoform of MMP-2 is the cause or the result of diabetic cardiomyopathy, further direct or indirect suppression studies should be conducted.

In conclusion, two isoforms of MMP-2 were expressed strongly in diabetic cardiac cells and tissue and those MMP-2 isoforms may be related to the functional changes in diabetes and structural changes by extension in the more advanced diabetic models.

## Supporting information

S1 Fig Histologic change in diabetic mouse hearts at 24 weeks. a, b, c: H&E stained control and DM mouse sections. d, e, f: MT stained control and DM mouse sections. g, h, i: PAS stained control and DM mouse sections. j, k, l: TUNEL stained control and DM mouse sections. Compared to control and DM 12week groups, thickened cardiomyocytes with increased collagen accumulation and apoptosis were noted in H&E, MT, and TUNEL stain, respectively at DM 24 week group.
(TIF)
