# Supplementary figures and images for: Enhanced cardiac expression of two isoforms of matrix metalloproteinase-2 in experimental diabetes mellitus
Source: PLoS One. 2019 Aug 28;14(8):e0221798. doi: 10.1371/journal.pone.0221798 (PMC6713391; doi:10.1371/journal.pone.0221798)

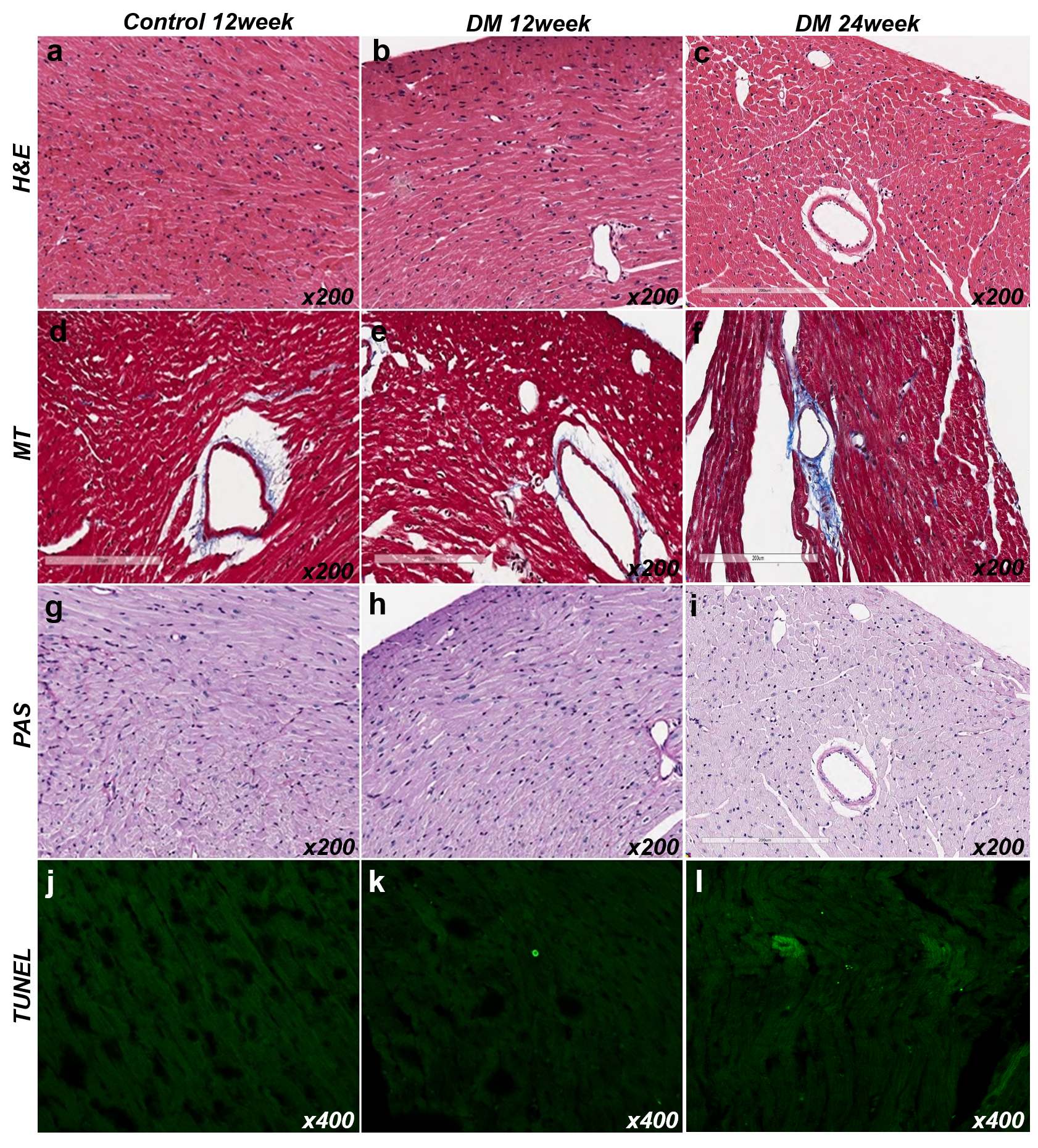

Supplement: S1 Fig — a, b, c: H&E stained control and DM mouse sections. d, e, f: MT stained control and DM mouse sections. g, h, i: PAS stained control and DM mouse sections. j, k, l: TUNEL stained control and DM mouse sections. Compared to control and DM 12week groups, thickened cardiomyocytes with increased collagen accumulation and apoptosis were noted in H&E, MT, and TUNEL stain, respectively at DM 24 week group. (TIF) [file pone.0221798.s001.tif]
